# The Road to Hell Is Paved with Good Intentions: Why Harm–Benefit Analysis and Its Emphasis on Practical Benefit Jeopardizes the Credibility of Research

**DOI:** 10.3390/ani7090070

**Published:** 2017-09-11

**Authors:** Herwig Grimm, Matthias Eggel, Anna Deplazes-Zemp, Nikola Biller-Andorno

**Affiliations:** 1 Messerli Research Institute, University of Veterinary Medicine, 1210 Vienna, Austria; herwig.grimm@vetmeduni.ac.at; 2 Institute for Biomedical Ethics and History of Medicine, University of Zurich, 8006 Zurich, Switzerland; deplazes@ethik.uzh.ch (A.D.-Z.); biller-andorno@ethik.uzh.ch (N.B.-A.)

**Keywords:** harm–benefit-analysis, legitimacy of animal research, value of knowledge, scientific vs. societal benefit, animal ethics

## Abstract

**Simple Summary:**

The European legislation on project evaluation of animal research has recently changed. Every procedure on live non-human vertebrates and cephalopods has to be approved in a project evaluation (harm–benefit analysis (HBA)) that weighs the inflicted harms on animals against potential prospective benefits. Recent publications on the HBA prioritise “societal benefits” that have a foreseeable, positive impact on humans, animals, or the environment over gaining knowledge (e.g., basic research). However, we argue that whether potential prospective societal benefits are realized is (a) impossible to predict and (b) exceeds the scope and responsibility of researchers. Furthermore, the emphasis on practical benefits has the drawback of driving researchers into speculation on the practical benefit of their research and, therefore, into promising too much. Repeated failure to deliver proclaimed practical benefits will lead to a loss of trust and credibility in research. The concepts of benefit and benefit assessment in the HBA, as well as the HBA itself, require re-evaluation in a spirit that embraces the value of knowledge in our society. Research projects should be measured by the quality of the research they perform and by the contributions they make to a specific field of research or research program. Only then can promises regarding benefits (in terms of knowledge) be kept and continued public trust ensured. Time and again, scientific knowledge has been utilized to great benefit for humans, animals, and the environment. The HBA, as it currently stands, tends to turn this idea upside down and implies that research is of value only if the resulting findings bring about direct practical benefits, which science itself can neither provide nor guarantee. The road to hell is, as the saying goes, paved with good intentions.

**Abstract:**

It is our concern that European Union Directive 2010/63/EU with its current project evaluation of animal research in the form of a harm–benefit analysis may lead to an erosion of the credibility of research. The HBA assesses whether the inflicted harm on animals is outweighed by potential prospective benefits. Recent literature on prospective benefit analysis prioritizes “societal benefits” that have a foreseeable, positive impact on humans, animals, or the environment over benefit in the form of knowledge. In this study, we will argue that whether practical benefits are realized is (a) impossible to predict and (b) exceeds the scope and responsibility of researchers. Furthermore, we believe that the emphasis on practical benefits has the drawback of driving researchers into speculation on the societal benefit of their research and, therefore, into promising too much, thereby leading to a loss of trust and credibility. Thus, the concepts of benefit and benefit assessment in the HBA require a re-evaluation in a spirit that embraces the value of knowledge in our society. The generation of scientific knowledge has been utilised to great benefit for humans, animals, and the environment. The HBA, as it currently stands, tends to turn this idea upside down and implies that research is of value only if the resulting findings bring about immediate societal benefit.

## 1. Introduction

European Union Directive 2010/63/EU, which has recently been transposed into the legislation of all EU member states, explicitly addresses the public’s increased ethical concerns for animals and reflects the widespread attribution of moral status to animals in Western societies. “Animals have an intrinsic value which must be respected. There are also the ethical concerns of the general public as regards the use of animals in procedures. Therefore, animals should always be treated as sentient creatures and their use in procedures should be restricted to areas, which may ultimately benefit human or animal health, or the environment” [1] (recital 12). To ensure compliance with these standards and officially validate scientific research in the face of ethical scrutiny, the directive aims to provide for an unbiased, consistent, comprehensive, and transparent framework for project evaluation: Every project proposal in EU member states pursuing one of the defined scientific purposes in Art. 5 of the Directive that entails procedures on live animals (i.e., non-human vertebrates and cephalopods [1] (Art.) has to undergo a review. This project evaluation [1] (Art. 38) includes compliance to the 3Rs [2] to ensure that animals are only used if there are no less harmful or alternative methods available. Besides other criteria, every project proposal has to undergo a harm–benefit analysis (HBA) designed “to assess whether the harm to the animals in terms of suffering, pain and distress is justified by the expected outcome taking into account ethical considerations, and may ultimately benefit human beings, animals or the environment” [1] (Art. 38d). While this passage implies that some sort of benefit needs to be at stake to validate harm inflicted on animals, its wording lacks precision, and nowhere are its terms “benefit” and “ethical consideration” clearly defined [3]. In practice, responsibility for carrying out the HBA falls on national authorities and their evaluations often draw on the expertise of international working groups [4]. Relevant literature on the prospective benefits of animal research prioritises “practical benefits” that have a foreseeable, positive impact on humans, animals, or the environment [5,6,7,8,9]. The tendency in recent publications has been to view translational, applied research and practical benefits as weightier than benefits that come in the form of adding to our knowledge base or developing research methods, which are precisely the benefits of basic research [4,5,10,11,12,13,14]. Recent analyses of German law, for instance, demonstrate that this normative position has indeed found its way into national legislation, e.g., in Bremen, Germany, Andreas Kreiter’s license to work on macaques was not renewed because his work was “too far from applications” [15] and because “it is ethically not justifiable to inflict this kind of pain on animals for the generation of neurobiological basic knowledge” [16] (Authors’ note: Translation ours). It is important to note that the formulation of the Directive leaves room for interpretation and thus, the understanding of the HBA in the literature [5,6,7,8,9,10] and the strong claims that follow from it are not necessarily in the Directive’s intention.

It is our concern that the HBA in its present form and implementation, although formulated with the best of intentions, may lead to an erosion of the credibility of research.

## 2. Understanding “Benefit” in the HBA

Generally speaking, the idea of the HBA builds on a consequentialist moral theory and brings it into a legal framework. Consequentialist theories allow for justifying harms on the basis of expected benefits. In other words, animal interest can be sacrificed if significant interests of others are met [17]. In consequence, the question emerges of which benefits are significant and of relevance in the HBA. Directive 2010/63/EU frames the term “benefit” in the context of a potential to ultimately benefit humans, animals, or the environment [1] (Article 38 (2)). The *Expert Working Group on Project Evaluation and Retrospective Assessment* identifies “What?”, “Who?”, “How?”, and “When?” as key questions for estimating benefits in a HBA in an official guiding document commissioned to facilitate the implementation of the directive at member state level [4]. As we read this document, the Expert Working Group seems to be suggesting that the selling point of animal research is a practical benefit. However, the actual expected outcome of animal research is and can only ever be data. A single research project can produce findings that are necessary but, on their own, never sufficient for attaining (promised) benefits. Take, for instance, the routinely used promise of drug development as an example of high benefits. First, one single project by itself hardly ever leads to a new medication. It is rather a series of experiments and projects that build on the knowledge from one another. Second, whether the findings of one experiment will actually bring about new medication or disease treatment does not hinge on those findings alone, regardless of how well-designed and thoroughly carried out the experiment is. It also depends on competing drugs on the market, profitable manufacturing, efficient distribution, etc. The findings of an experiment can only be necessary but never sufficient conditions for the development of a new treatment. Of course, data obtained through research is often used to create benefits, but explicit benefits are impossible to predict and thus belong to what the working group coined “promise dimension” [14] of animal research. It is possible that potential societal benefits of whole research fields—not single projects—may be more accurately predicted had we better retrospective analyses of former projects. With regard to this problem, studies such as Williams et al. [18] or Comroe and Dripps [19] showing the number of research projects that together led to medical advancement are important. Furthermore, studies such as “Project Retrosight” [20] not only analyse what knowledge and benefits were generated from research projects but also what factors are associated with the generation of knowledge and benefit. However, there are not enough robust systematic reviews [21,22] of medical achievements of animal research projects and thus additional systematic reviews would be very important in this context.

Directive 2010/63/EU stipulates that harm to live research animals needs to be justified by an expected outcome. Whether that harm may ultimately benefit human beings, animals or the environment needs to be estimated in advance. However, as explained above, whether practical benefits are realized is (a) impossible to predict and (b) exceeds the scope and responsibility of researchers. Of course, one could argue optimistically—or quite cynically—that nobody can by any means exclude the possibility that any research project *may ultimately* benefit human beings, animals, or the environment, but that can hardly be the intent of the directive, since every project would have to be approved. Furthermore, even if research *may ultimately* benefit someone or something, the question remains: What constitutes a valid benefit? Existing literature [4,10,11,12,13] and reviews thereof [5,14] quite obviously regard “practical benefits” as having greater legitimating power than scientific benefits (e.g., expanding the knowledge base) [6,8,9].

## 3. The Promise of Practical Benefit

If some projected benefit is to remain the crucial factor in a HBA, and the expectancy prevails that this benefit be practical in nature, one logical consequence is that applied and translational research—which supposedly aim at practical benefits—will outweigh and possess more legitimating power than basic research. This would mirror the common bias towards applied and translational research in present interpretations of the HBA. However, applied and translational research are no more capable than basic research of yielding anything more than knowledge that is a necessary but never sufficient condition of practical benefits.

Furthermore, the emphasis on practical benefits presses the applicant to identify some expected practical benefits of her research so as to maximise the likelihood of a favorable project evaluation. Pressure of that nature has the drawback of driving researchers into speculation on the practical benefit of their research and, therefore, into promising too much. This problem exacerbated by an obligation to make the non-technical project summary publicly available in which the harms and benefit of the project have to be indicated. Yet, if researchers choose to make practical benefits their selling point, they are bound to fail. Repeated failure to delivering on stated promises of practical benefits will lead science into a loss of credibility. When challenged with the question of whether the promises outlined in project proposals and applications have been fulfilled, no research will ever be able to confirm its success and value through documented practical benefits. This will neither lead to increased public trust in nor strengthen the ethical credibility of animal research, but quite the opposite: loss of trust and credibility are the very serious and probable consequences of repeated failure to produce proclaimed practical benefits. An ensuing decrease in public and financial support is equally foreseeable.

To not overstate the limitations of the HBA, we would like to shortly summarize and clarify which flaws we believe are inherent to the process and which flaws could be overcome:

First, we argue that prospective benefit assessment of one project is inherently afflicted with a very high degree of uncertainty. Thus, we believe that prospective assessment of the potential knowledge generated by a project would be much more plausible. Therefore, in project evaluation, we suggest focussing on the potential knowledge of a project, rather than its potential benefits.

Second, it can take hundreds of projects for a benefit to be realised [18,19], thus prospective benefit assessment might be more plausible for whole research fields, rather than single projects. Unfortunately, to date, we lack sufficient systematic retrospective analysis on what benefit research fields have generated in the past. By putting more emphasis on retrospective benefit assessment, we might be able to improve future prospective benefit assessments of research fields.

Third, the problem of promising too much in non-technical project summaries of project proposals could be overcome by shifting the focus from promises of almost immediate practical benefits to better explanations to the public about the expected knowledge gain of a project and its importance for a research field. This, of course, is only possible if knowledge is regarded as a valuable and legitimate scientific outcome in project evaluation as well as in the eye of the public.

## 4. Conclusions

Historically, the professional self-regulation granted to scientific research mirrored the public’s general trust in science, its goals and achievements. This autonomy is now challenged by the requirement to legitimise research through practical benefits in a HBA. We believe that harms to animals must ultimately be justified by benefits. However, we advise caution in the operationalization of benefit in terms of direct/immediate, long-term or guaranteed benefit. It is important to realize that research programs may or may not yield practical benefits for humans, animals or the environment, and that this lack of immediately and clearly foreseeable practical benefit in a research program does not indicate bad science. The honest and transparent acknowledgment that good science produces good data and not benefits would be a first step toward building and/or regaining credibility.

The concepts of benefit and benefit assessment in the HBA, as well as the HBA itself, require re-evaluation in a spirit that embraces the value of knowledge in our society. As we argued, the “value of knowledge” can be understood in two relevant ways: (a) *scientific value of knowledge*, qualified by its relative importance and contribution to a given research field; (b) *societal value of knowledge*, qualified by its potential to allow for benefits which are of importance for a given society. Research projects should be measured by the quality of the research they perform and by the contributions they make to a specific field of research or research program. Only then can promises regarding benefits (in terms of knowledge) be kept and continued public trust ensured. We do not advise that the standards of project evaluation be lowered or that more projects be carried out, nor do we propose a “carte blanche” for basic research and research in general. 

On the contrary, our argument is that appropriate criteria within the capacity of the applying researchers (e.g., scientific rigor, proper statistics, sound methods) be enforced. However, whether scientific standards in animal research are met is currently questioned [23,24]. It is the researchers’ responsibility that only well-designed projects that meet the relevant scientific standards should be carried out to maximize scientific benefit (knowledge) and consequently potential societal benefit. Here, systematic reviews could help identify detrimental factors such as bias, poor study design and poor reporting methods, among others and thus could help improve scientific standards [21] and thus maximize epistemic benefit of animal research. Failing to meet scientific standards together with not meeting self-proclaimed goals (societal benefit), would further exacerbate the erosion of trust in science.

An important question remains: how to decide which research fields are of relevance and can justify the use of animals. In our view, this question can only be dealt with on a political level and not in a project evaluation. In this arena, it should be decided which research we, as a society, deem important enough to use animals and which research we deem worthy of being funded by tax payers.

Contributing to the knowledge base with new findings that are meaningful and important for understanding our world has been a pillar of scientific success in the past. Time and again, scientific knowledge has been utilised to great benefit for humans, animals, and the environment. The HBA, as it currently stands, tends to turn this idea upside down and implies that research is of value only if the resulting findings bring about direct practical benefits, which science itself can neither provide nor guarantee. The road to hell is, as the saying goes, paved with good intentions.

## References

[1] European Commission (2010). Directive 2010/63/EU of the European Parliament and of the Council of 22 September 2010 on the protection of animals used for scientific purposes. Off. J. Eur. Union.

[2] Russell W.M.S., Burch R.L. (1959). The Principles of Humane Experimental Technique.

[3] Grimm H. (2015). Turning apples into oranges? The harm–benefit analysis and how to take ethical considerations into account. Altern. Lab. Anim..

[4] Working Document on Project Evaluation and Retrospective Assessment. http://ec.europa.eu/environment/chemicals/lab_animals/pdf/Endorsed_PE-RA.pdf.

[5] Alzmann N. (2016). Zur Beurteilung der Ethischen Vertretbarkeit von Tierversuchen.

[6] Porter D.G. (1992). Ethical scores for animal experiments. Nature.

[7] Scharmann W., Teutsch G.M. (1994). Zur ethischen Abwägung von Tierversuchen. ALTEX.

[8] Stafleu F.R., Tramper R., Vorstenbosch J., Joles J.A. (1999). The ethical acceptability of animal experiments: A proposal for a system to support decision-making. Lab. Anim..

[9] Bout H.J., van Vlissingen J.M.F., Karssing E.D. (2014). Evaluating the ethical acceptability of animal research. Lab. Anim..

[10] Ach J.S., Borchers D., Luy J. (2009). Zur ‘ethischen Vertretbarkeit’ von Tierversuchen. Der Ethisch Vertretbare Tierversuch.

[11] Imboden M.D., Sigg H., Folkers G. (2011). Über die Grundlagenforschung und den Wert der Erkenntnis. Güterabwägung bei der Bewilligung von Tierversuchen.

[12] Abbot A. (2010). Basel declaration defends animal research. Nature.

[13] Laber K., Newcomer C.E., Decelle T., Everitt J.I., Guillen J., Brønstad A. (2016). Recommendations for Addressing Harm–Benefit Analysis and Implementation in Ethical Evaluation—Report from the AALAS–FELASA Working Group on Harm–Benefit Analysis—Part 2. Lab. Anim..

[14] Brønstad A., Newcomer C.E., Decelle T., Everitt J.I., Guillen J., Laber K. (2016). Current concepts of Harm–Benefit Analysis of Animal Experiments—Report from the AALAS–FELASA Working Group on Harm–Benefit Analysis—Part 1. Lab. Anim..

[15] Schiermeier Q. (2008). German Authority halts primate work. Nature.

[16] Oberverwaltungsgericht Bremen 2013:669. http://www.oberverwaltungsgericht.bremen.de/sixcms/detail.php?gsid=bremen72.c.11099.de&asl=bremen72.c.11265.

[17] Persson K., Elger B.S., Shaw D.M. (2017). The Indignity of Relative Concepts of Animal Dignity: A Qualitative Study of People Working with Nonhuman Animals. Anthrozoös.

[18] Williams S.R., Lotia S., Holloway A.K., Pico A.R. (2015). From scientific discovery to cures: Bright stars within a galaxy. Cell.

[19] Comroe J.H., Dripps R.D. (1974). Ben Franklin and open heart surgery. Circ. Res..

[20] Project Retrosight. https://www.rand.org/pubs/periodicals/health-quarterly/issues/v1/n1/16.html.

[21] CAMARADES. http://www.dcn.ed.ac.uk/camarades/default.htm#about.

[22] Perel P., Roberts I., Sena E., Wheble P., Briscoe C., Sandercock P., Macleod M., Mignini L.E., Jayaram P., Khan K.S. (2007). Comparison of treatment effects between animal experiments and clinical trials: Systematic review. Br. Med. J..

[23] Vogt L., Reichlin T.S., Nathues C., Würbel H. (2016). Authorization of animal experiments is based on confidence rather than evidence of scientific rigor. PLoS Biol..

[24] Reichlin T.S., Vogt L., Würbel H. (2016). The researcher’s view of scientific rigot—Survey on the conduct and reporting of in vivo research. PLoS ONE.

